# Cancer-Related Triplets of mRNA-lncRNA-miRNA Revealed by Integrative Network in Uterine Corpus Endometrial Carcinoma

**DOI:** 10.1155/2017/3859582

**Published:** 2017-02-08

**Authors:** Chenglin Liu, Yu-Hang Zhang, Qinfang Deng, Yixue Li, Tao Huang, Songwen Zhou, Yu-Dong Cai

**Affiliations:** ^1^School of Life Sciences and Biotechnology, Shanghai Jiaotong University, Shanghai 200240, China; ^2^Institute of Health Sciences, Shanghai Institutes for Biological Sciences, Chinese Academy of Sciences, Shanghai 200031, China; ^3^Department of Medical Oncology, Shanghai Pulmonary Hospital, Cancer Institute, Tongji University Medical School, Shanghai 200433, China; ^4^Key Lab of Computational Biology, CAS-MPG Partner Institute for Computational Biology, Shanghai Institutes for Biological Sciences, Chinese Academy of Sciences, Shanghai 200031, China; ^5^Collaborative Innovation Center for Genetics and Development, Fudan University, Shanghai 200433, China; ^6^School of Life Sciences, Shanghai University, Shanghai 200444, China

## Abstract

The regulation of transcriptome expression level is a complex process involving multiple-level interactions among molecules such as protein coding RNA (mRNA), long noncoding RNA (lncRNA), and microRNA (miRNA), which are essential for the transcriptome stability and maintenance and regulation of body homeostasis. The availability of multilevel expression data enables a comprehensive view of the regulatory network. In this study, we analyzed the coding and noncoding gene expression profiles of 301 patients with uterine corpus endometrial carcinoma (UCEC). A new method was proposed to construct a genome-wide integrative network based on variance inflation factor (VIF) regression method. The cross-regulation relations of mRNA, lncRNA, and miRNA were then selected based on clique-searching algorithm from the network, when any two molecules of the three were shown as interacting according to the integrative network. Such relation, which we call the mRNA-lncRNA-miRNA triplet, demonstrated the complexity in transcriptome regulation process. Finally, six UCEC-related triplets were selected in which the mRNA participates in endometrial carcinoma pathway, such as CDH1 and TP53. The multi-type RNAs are proved to be cross-regulated as to each of the six triplets according to literature. All the triplets demonstrated the association with the initiation and progression of UCEC. Our method provides a comprehensive strategy for the investigation of transcriptome regulation mechanism.

## 1. Introduction

Uterine corpus endometrial carcinoma (UCEC) develops from the cells of the inner lining of the uterus, which is one of the most common female genital cancer threatening the health of women all over the world [1, 2]. Only counting 2012, approximately 320,000 women have been diagnosed and about 76,000 people have died of UCEC, according to incomplete statistics [3]. Most commonly, UCEC occurs in postmenopausal women, due to the unstable level of estrogen after menopause [4]. Smoking, high blood pressure, and being overweight also indirectly relate to uterus diseases via various regulation mechanisms [5–7]. In addition, genetic disorders also contribute to the development of UCEC and associate it with other diseases such as Lynch syndrome and colon cancer [8, 9]. A potential inherited tendency shows in UCEC with an increased risk in women with a family history of endometrial cancer [10]. Clinical diagnosis is according to the symptoms such as postmenopausal vaginal bleeding, enlarged uterus, low abdominal pain, and pelvic cramping [11–13]. The understanding of regulatory network could help to investigate its mechanism and benefit the diagnosis and treatment of UCEC.

The multi-type-molecular regulatory network, especially the interaction network between coding RNAs (mRNAs) and noncoding RNAs, has gained many interests in recent years. Previous reports have revealed that the microRNAs (miRNAs) which are small size noncoding RNAs of about 22 nucleotides and long noncoding RNAs (lncRNAs) which contain more than 200 nucleotides cross-regulate their expression levels and comodulate the expression of mRNAs. On the other hand, mRNAs also affect the expression of noncoding RNAs in specific ways [14, 15]. For example, the long intergenic noncoding RNA lincRNA-p21 has been reported to be downregulated by miRNA let-7. The binding of lincRNA-p21 to JUNB and CTNNB1 mRNAs results in the repression of JunB and *β*-catenin translation [16]. Another experiment has shown that the depletion of lncRNA highly upregulated in liver cancer (HULC) results in significant deregulation of several genes involved in liver cancer. This lncRNA is upregulated by CREB mRNA which is underregulated by miR372 [17]. Such interaction, which we call the mRNA-lncRNA-miRNA triplet, is essential for the maintenance and regulation of body homeostasis. The aberrance of any of its molecules may influence the stability of multilevel expression and affect the tumorigenesis accordingly.

Recently, the availability of large scaled multilevel expression data provides an opportunity to obtain the comprehensive map of the multi-type-molecular regulatory network. The Cancer Genome Atlas (TCGA) database [18–20], especially the TCGA long noncoding RNAs website, provides the whole-genome profiling of 301 UCEC patients including the expression levels of mRNA, lncRNAs, and miRNAs. Such multidimensional resources allow us to investigate the mRNA-lncRNA-miRNA interactions, understand the transcriptional characteristic of UCEC, and dig deeper into the essential genetic alterations, transcriptional regulations, and posttranscriptional mechanisms throughout its initiation and progression [19].

Here, a new method is built to systematically investigate the mRNA-lncRNA-miRNA interactions in UCEC based on the patient expression profiles downloaded from TCGA long noncoding RNA website. An integrative network of mRNAs, lncRNAs, and miRNAs is constructed using an accurate and extremely efficient algorithm, the variance inflation factor (VIF) regression method. Many mRNA-lncRNA-miRNA triplets, which depict the cross-regulation relations among mRNA, lncRNA, and miRNA, are detected by searching all cliques (that is complete subgraphs with all vertices adjacent to each other) consisting of these three elements. The clique searching problem is a fundamental topic in computer science, which is very important in clustering analysis based on density and grid of data elements [21], and many solutions have been proposed to improve the searching performance. At last, the detected triplets are screened for their biological functions, and three of them are determined as UCEC-related triplets according to KEGG database and published literature. All in all, the proposed algorithm can find out disease-associated transcriptional RNA (miRNAs, lncRNAs, and mRNAs) interactions and may contribute to reveal the potential posttranscriptional regulatory mechanisms of UCEC.

## 2. Material and Methods

### 2.1. Datasets

The expression data of UCEC are obtained from TCGA long noncoding RNAs website (http://larssonlab.org/tcga-lncrnas/datasets.php), including the profiles of 20,462 protein coding genes, 10,419 lncRNA genes, and 742 miRNAs from 301 UCEC patients, as shown in Table 1. Specifically, the miRNA expression data are selected from the profiles of noncoding genes by their gene symbols. The expression levels are given as reads per kilobase per million (RPKM) values. The zero values of the expression data are set as the minimum nonzero RPKM of their corresponding sample for the allowance of log transformation. UCEC-related pathway information is adopted from KEGG (Kyoto Encyclopedia of Genes and Genomes, http://www.kegg.jp/) database with entry ID “hsa05213”. The pathway involves 52 genes including tumor protein coding gene TP53 and cadherin protein coding gene CDH1.

### 2.2. Integrative Network Construction

The interaction network is built by firstly determine the key factors (mRNA, lncRNA, and miRNA) affecting the expression level of each RNA molecule, respectively, and integrating them into a complete network after that. Hence, each RNA molecule is regarded as the dependent variable in one linear regression model, while all others are treated as the independent variables. In summary, 20,462 + 10,419 + 742 = 31,623 regression models are built where each one is based on 31,622 RNA expression features, and the integrative network is constructed after that.

Due to the large dataset in each regression model with far more features than observations (31,622 versus 301), an efficient regression and feature selection method, the variance inflation factor (VIF) regression algorithm [22] is utilized to select the optimal regulator set that is most related to each target RNA. The algorithm is designed to find the optimal *β* that can minimize the *l*_0_ penalized sum of squared errors,

(1) $$\begin{array}{c} \underset{\beta }{arg\;min}\left\{\;{\left\|\;y-X\beta \;\right\|}_{2}^{2}+\lambda_{0}{\left\|\;\beta \;\right\|}_{l_{0}}\;\right\}, \end{array}$$

where **y** = (*y*_1_,…,*y*_*n*_)′ are *n* observations and **X** = (**x**_1_,…**x**_*p*_) are *p* predictors, *p* ≫ *n*, ‖**β**‖_*l*_0__ = ∑_*i*=1_^*p*^*I*_{*β*_*i*_ ≠ 0}_. Instead of searching over all 2^*p*^ subsets for the best *β*, this algorithm evaluates the marginal correlations of each candidate predictors with the target factor using a small presampled set of data and searches the optimal subset by including *t*-statistic correction procedure when adding or removing one variable at a time. The method has shown great efficiency but is also accurate compared to other methods such as LASSO and has comparable accuracy even compared to the most accurate but slowest regression method FoBa. The construction of the VIF regression models is based on program from http://cran.r-project.org/web/packages/VIF/.

Next, the goodness-of-fit for linear regression models is assessed by the adjust coefficient of determination (denoted as adjust *R*^2^). The statistic measures how well the regression line approximates the real data points and can compare the regression model containing different number of regulators. In this paper, the regression model is retained only if it surpasses the adjust *R*^2^ cutoff of 0.8. Regulation relations between the target RNA and its regulators are obtained from the retained regression models. These relations are further integrated as a comprehensive map for the mRNA-lncRNA-miRNA interaction. Note that the constructed network is an undirected graph. The edges are constructed if the two factors are connected by arcs of any direction. It is because the regression model can only identify the regulators of the target based on their gene expression associations but cannot determine if the regulators induce the perturbation of the target or vice versa without prior biological knowledge.

### 2.3. The mRNA-lncRNA-miRNA Triplet Detection

The detection of mRNA-lncRNA-miRNA triplets from the integrative network is a typical clique problem in computer science. Clique problem tries to search all complete subgraphs with all vertices connected to each other. Here, the size of subgraph is set as three, and the vertices of each subgraph are restricted to contain all of the three RNA types. The subgraphs, called mRNA-lncRNA-miRNA triplets, describe the relations of mRNA, lncRNA, and miRNA with each two of them coregulated according to the VIF regression model. The detection procedure is fulfilled by the “cliques” function in R package* igraph* [23].

Next, the UCEC-related triplets are further screened out if its mRNA participates in the hsa05213 pathway (endometrial cancer, Homo sapiens) according to KEGG database. These triplets are further analyzed for their interactions and biological functions as to UCEC according to literature.

## 3. Results and Discussion

### 3.1. Structure of the Integrative Network

The whole-genome integrative network of mRNA, lncRNA, and miRNA is constructed based on their cross-regulation relations using VIF regression. Totally, 19,098 factors are included in the integrative network, composed of 14229 coding mRNAs, 4,601 lncRNA, and 268 miRNA. On the network, each RNA is regulated by an average of 30 factors. The protein coding gene-gene interactions dominate the integrative network, as the expression levels of most coding genes are largely affected by only the coding mRNAs. Noncoding RNAs tend to have more interactions with noncoding RNAs instead of coding RNAs, which implies the extensive cross-talk of noncoding RNAs in their regulation of transcriptome and posttranscriptome. The details of the integrative network can be referred in Supplementary Material S1 available online at https://doi.org/10.1155/2017/3859582.

### 3.2. Candidate mRNA-lncRNA-miRNA Triplets of UCEC

By restricting the vertices types of clique problem to have all three RNA types, 14,416 mRNA-lncRNA-miRNA triplets are detected from the integrative network. These triplets involve 736 coding mRNAs, 1,799 lncRNAs, and 227 miRNAs, and provide a comprehensive map for the mRNA-lncRNA-miRNA interaction. The relatively small number of coding mRNAs compared to the noncoding RNAs indicates that many coding genes are coregulated by multiple lncRNAs and miRNAs. Extensive cross-talks exist in the regulatory process of noncoding RNAs, which also explain the complexity of transcriptome regulation process. The list of the detected triplets can be found in Supplementary Material S2.

**Figure 1:**
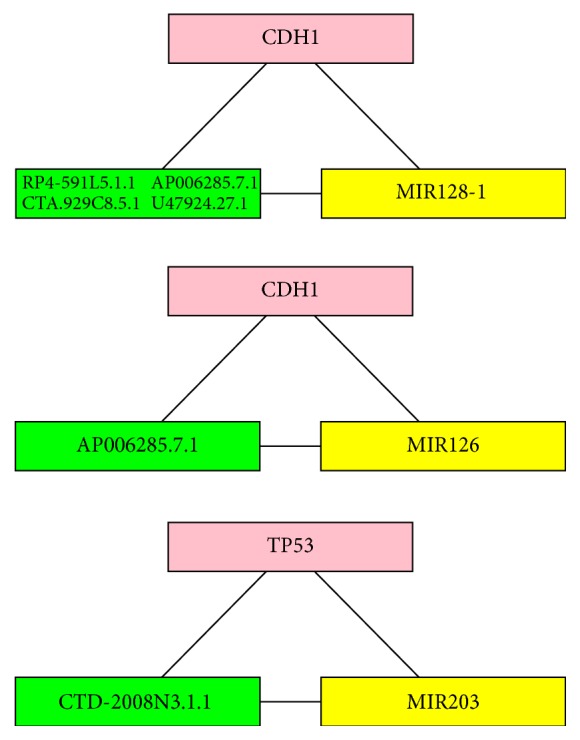
UCEC-related triples screened out from the integrative network. The mRNA, lncRNA, and miRNA are labeled as red, green, and yellow. The cross-regulation relations are described as an undirected 3-vertex graph. The four triplets including the same mRNA and miRNA but different lncRNAs are presented in the top figure. The other two triplets are presented in the following.

Next, the triplet is considered as UCEC-related if its mRNA participates in hsa05213 endometrial cancer pathway. Note that the mRNA-lncRNA-miRNA cross-interaction is a very special interaction case that the genes in the selected triplet have little chance to be enriched in the pathway. However, studies have shown that the mutations in a pathway are mutual exclusive, and only one functional gene mutation is enough to perturb the pathway [24–26]. Hence, any triplet having overlapped genes with the pathway may contribute to the progression of cancer. Here, six triplets related to hsa05213 are detected and are retained for further analysis, as shown in Figure 1. The mRNA, lncRNA, and miRNA are labeled as red, green, and yellow, and the cross-regulation relations are shown as an undirected 3-vertex graph. Four of the six triplets, as shown in the first graph in Figure 1, involve the same mRNA and miRNA, but different lncRNAs, that is, mRNA CDH1-lncRNA (RP4-591L5.1, CTA.929C8.5.1, U47924.27.1, and AP006285.7.1)-miRNA miR128-1. The other two triplets are mRNA CDH1-lncRNA AP006285.7.1-miRNA miR126 and mRNA TP53-lncRNA CTD-2008N3.1.1-miRNA miR203, respectively.

### 3.3. Interaction and Biological Function of UCEC-Related Genes and Triplets

First, we focus on the mRNA-miRNA interaction and biological functions of the first set of triplets in Figure 1, which involves CDH1 and miR128-1, as mentioned above. CDH1 encodes a classical cadherin from cadherin superfamily, which is a calcium-dependent cell adhesion regulatory protein [27, 28]. CDH1 contributes the cell adhesion, mobility, and proliferation in specific microenvironment, especially in tumor [29]. As for UCEC, CDH1 contributes the initiation and invasion of endometrial cancer through its specific role in epithelial-mesenchymal transition (EMT) [30–32]. Additionally, miR128-1 has been proved to interact with the expression product of CDH1 cadherin and participate in the regulation of EMT in prostate cancer stem during the tumorigenesis [33, 34]. Apart from that, miR128-1 also participates in the regulation of progression and EMT in glioblastoma [35]. In fact, miR128-1 interacts with CDH1 coding protein cadherin via a specific upstream protein Bmi1 which is the direct target of miR128-1 [34, 35].

Next, we consider the four lncRNAs in the triplets. The first lncRNA RP4-591L5.1 is a crucial lncRNA which binds a specific miRNA miR218. MiR218 contributes the cellular chemosensitivity, migration, and invasion, which may further influence the cadherin regulation and associate with the function of miR128-1 [36, 37]. Another lncRNA, CTA.929C8.5.1, also called lnc-CRYBA4-7:1, has been predicted to be interacted with miR4268. MiR4268 is a rare miRNA with a special 3D structure. It has been proved to participate in the maintenance of stemness and may activate the initiation process of tumor in specific environment [38]. Additionally, as the stemness of cancer cells is associated with tumor migration and has specific relationship with the process of EMT [39, 40], the interaction of CTA.929C8.5.1 with miR4268 may affect the stemness of tumor cells and further have a specific influence on EMT, which explains its potential relationship with miR218 and gene CDH1 in the triplet. The third lncRNA U47924.27.1, also named lnc-PTPN6-1:1, is a unique lncRNA that is the target of several functional miRNAs, such as miR139, and may participate in the initiation of several tumors especially in hematopoietic malignancy [41]. Therefore, it is reasonable that, in UCEC, such lncRNA may play a similar way to interact with the miRNA and lncRNAs mentioned above and contribute to the tumor initiation and progression. Apart from that, U47924.27.1 is associated with PTEN, while CDH1 coding protein cadherin is also associated with PTEN cascades. Hence, this lncRNA may also have interaction with CDH1 [42]. The last lncRNA AP006285.7.1, also annotated as lnc-KRTAP5-4-1:1, has been proved to be associated with miR513a through sequence analysis. MiR513a may play its specific role in various cancer types and mainly regulate the proliferation of cells and contributes to the modeling of inflammation environment [43, 44]. Such regulatory functions may participate in EMT process and further promote the migration of tumor cells, which further explains the interaction of AP006285.7.1 with other factors in the triplet. In summary, all elements in the triplet contribute cohesively to the initiation and progression of UCEC and may exert influence on EMT process.

As to the triplet CDH1-lncRNA AP006285.7.1-miRNA miR126, we only investigate the functions of miR126 since the other two have been mentioned above. MiR126 has been proved as a predictive and diagnosis marker of esophageal cancer [45]. It is also associated with cell adhesion and migration and may contribute to the cadherin regulation in a similar way with other miRNAs (miR99a, miR200, etc.) [46, 47]. Therefore, miR126, lncRNA AP006285.7.1, and CDH1 can be clustered together because of their specific function and contribution to UCEC. This triplet focuses more on the cell adhesion instead of EMT progression and concentrates on the progression and migration process of the tumorigenesis of UCEC.

The next triplet is mRNA TP53-lncRNA CTD-2008N3.1.1-miRNA miR203. TP53 is the most famous tumor suppressor gene which generally contributes to every common type of tumor including endometrial cancer [48, 49]. As a multifunctional gene, TP53 also interacts with several crucial miRNAs (miR181, miR34a, miR520g, etc.) especially in various tumor tissues [50–53]. Consistent with our screen triplet, the interaction of TP53 and miR203 has been proved by several publications [54, 55]. Such interaction is quite crucial for certain kind of tumor especially for colon cancer [55]. As to lncRNA CTD-2008N3.1.1, which is also called lnc-CTD-2012 M11.2.1-1:1, it has been reported to associate with several miRNAs using computational prediction, which may have its specific way to interact with TP53 and miR203 in UCEC [56–58]. Additionally, CTD-2008N3.1.1 interacts with miR331 which participates in the tumorigenesis of various tumor types [56, 59–61]. Furthermore, CTD-2008N3.1.1 is a specific lncRNA originating from CTD sequence [62]. Since TP53 has been reported to be associated with several CTD structures in different tumor types, such screened lncRNA may also interact with TP53 and have its specific function in the process of UCEC initiation and progression [63, 64].

## 4. Conclusion

In summary, all of the selected triplets have been partially or fully confirmed to be associated with tumorigenesis especially in UCEC. Moreover, some of our preliminarily screened genes which are not included in these six triplets are also proved to be UCEC-associated and may have their specific function in the process of tumorigenesis. For example, miR204 is a specific miRNA in non-small cell lung cancer, which can specifically regulate the metastasis of tumor cells [65]. Such miRNA may have similar function in UCEC. Another miRNA, miR320, has been reported as a functional regulatory miRNA in stage I endometrioid endometrial carcinoma [66]. All in all, based on the expression profile of UCEC, our proposed method can cluster miRNAs, lncRNAs, and coding genes into functional interacted groups. Such an algorithm can also be applied to other cancer types and benefit the deeper understanding of the transcriptome regulatory mechanisms, and the cross-talk of multilevel RNAs such as miRNAs and lncRNAs. Additionally, the transcriptional level regulation network prediction helps to reveal the posttranscriptional regulation in tumors and other severe diseases.

## Supplementary Material

Supplementary Material S1 Regulatory relations of the factors in constructed network. Supplementary Material S2 The triplet list for mRNA-lncRNA-miRNA cross-regulation.

## Figures and Tables

**Table 1:** Number of genes in raw data and constructed network of 301 UCEC patients.

	mRNA	lncRNA	miRNA	Total
Raw data	20,462	10,419	742	31,623
Network	14,229	4,601	268	19,098
Triplet list	736	1,799	227	2,762
